# Muc5b overexpression causes mucociliary dysfunction and enhances lung fibrosis in mice

**DOI:** 10.1038/s41467-018-07768-9

**Published:** 2018-12-18

**Authors:** Laura A. Hancock, Corinne E. Hennessy, George M. Solomon, Evgenia Dobrinskikh, Alani Estrella, Naoko Hara, David B. Hill, William J. Kissner, Matthew R. Markovetz, Diane E. Grove Villalon, Matthew E. Voss, Guillermo J. Tearney, Kate S. Carroll, Yunlong Shi, Marvin I. Schwarz, William R. Thelin, Steven M. Rowe, Ivana V. Yang, Christopher M. Evans, David A. Schwartz

**Affiliations:** 1 Department of Medicine, University of Colorado Denver, School of Medicine, Aurora, CO 80045 USA; 2 Department of Medicine, University of Alabama at Birmingham, School of Medicine, Birmingham, AL 35294 USA; 3 Marsico Lung Institute, University of North Carolina, Chapel Hill, NC 27599 USA; 4 Physics and Astronomy, University of North Carolina, Chapel Hill, NC 27599 USA; 5 Parion Sciences, Inc, Durham, NC 27713 USA; 6 Wellman Center for Photomedicine and Department of Pathology, Massachusetts General Hospital, Boston, Massachusetts; Department of Pathology, Harvard Medical School, Massachusetts General Hospital, Boston, Massachusetts, USA; 7 Department of Chemistry, The Scripps Research Institute, Jupiter, FL 33458 USA; 8 Department of Immunology, University of Colorado Denver, School of Medicine, Aurora, CO 80045 USA

## Abstract

The gain-of-function *MUC5B* promoter variant rs35705950 is the dominant risk factor for developing idiopathic pulmonary fibrosis (IPF). Here we show in humans that *MUC5B*, a mucin thought to be restricted to conducting airways, is co-expressed with surfactant protein C (*SFTPC*) in type 2 alveolar epithelia and in epithelial cells lining honeycomb cysts, indicating that cell types involved in lung fibrosis in distal airspace express *MUC5B*. In mice, we demonstrate that Muc5b concentration in bronchoalveolar epithelia is related to impaired mucociliary clearance (MCC) and to the extent and persistence of bleomycin-induced lung fibrosis. We also establish the ability of the mucolytic agent P-2119 to restore MCC and to suppress bleomycin-induced lung fibrosis in the setting of Muc5b overexpression. Our findings suggest that mucociliary dysfunction might play a causative role in bleomycin-induced pulmonary fibrosis in mice overexpressing Muc5b, and that MUC5B in distal airspaces is a potential therapeutic target in humans with IPF.

## Abstract (web summary)

The promoter variant rs35705950 confers a gain of function to the MUC5B gene and is the dominant risk factor for idiopathic pulmonary fibrosis. Here the authors show that mice overexpressing Muc5b in distal airspaces show impaired mucociliary clearance and increased susceptibility to bleomycin-induced lung fibrosis, and that both characteristics are reduced by treatment with a mucolytic agent.

## Introduction

Idiopathic pulmonary fibrosis (IPF) is a progressive fibrotic lung disease with a median survival of 3–5 years^1^ that worsens despite treatment^2,3^. A common gain-of-function *MUC5B* promoter variant rs35705950 is the dominant risk factor, either genetic or environmental, for the development of IPF^4,5^. MUC5B is a major gel forming mucin in the lung that plays a key role in mucociliary clearance (MCC) and host defense^5^ that is secreted from proximal submucosal glands and distal airway secretory cells^6–8^. The *MUC5B* promoter variant is associated with enhanced expression of the *MUC5B* transcript in lung tissue from unaffected subjects and patients with IPF^4,9^. In patients with IPF, excess MUC5B protein is especially observed in epithelial cells in the respiratory bronchiole and honeycomb cyst^7,8^, regions of lung involved in lung fibrosis. However, it remains unclear how MUC5B leads to the development of IPF.

Here we show that MUC5B is produced in cells lining distal airways and honeycomb cysts in human IPF. We also show that Muc5b overproduction in the distal lungs of mice are associated with MCC dysfunction and exaggerates the development of fibrosis, and that this can be prevented by treatment with a mucolytic agent. Our findings show a causative, dose-dependent role for Muc5b in murine lung fibrosis, and thus support development of mucolytic intervention strategies for human disease.

## Results

### MUC5B/Muc5b in the distal lung and effects on fibrosis

**Fig. 1:**
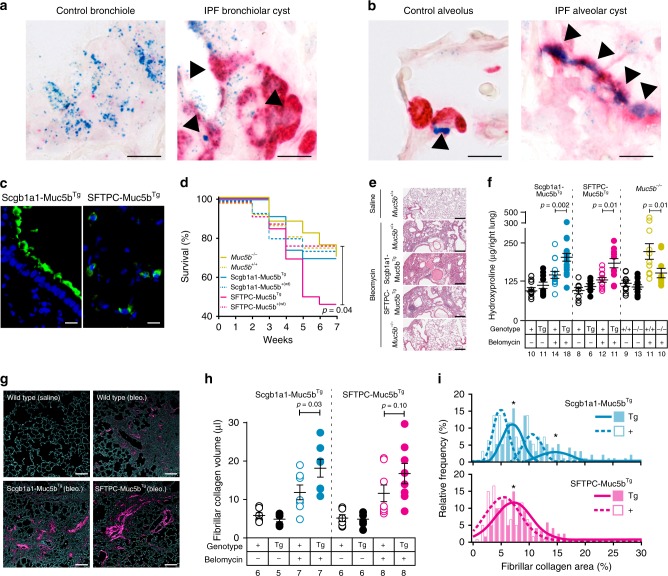
Muc5b overexpression in the distal lung is associated with greater fibrogenesis following bleomycin. **a**, **b** In situ hybridization of human lung specimens from control and IPF subjects with *MUC5B* variant rs35705950. Arrowheads depict cells co-expressing *SFTPC* (red) and *MUC5B* (blue) in control bronchioles and IPF bronchiolar structures **a** and in control type 2 alveolar epithelia and IPF type 2 alveolar structures **b**. **c** Transgenic mice expressing Muc5b under control of the mouse Scgb1a1 and the human SFTPC promoters demonstrate Muc5b protein in conducting airways and in type 2 alveolar epithelia. **d**, **e** After repeated doses of bleomycin (2.5 U/kg, IT on day 0, 1.5 U/kg on days 14 and 28), SFTPC-Muc5b^Tg^ demonstrated worse survival **d** and tissue injury **e** relative to wild type (+) littermates. Survival data in **d** were evaluated by *χ*^2^ statistic using 17 *Muc5b*^−/−^, 16 *Muc5b*^+/+^, 15 Scgb1a1- Muc5b^Tg^, 23 Scgb1a1-Muc5b^+(wt)^, 26 SFTPC- Muc5b^Tg^, and 25 SFTPC- Muc5b^+(wt)^, mice. **f**–**i** Scgb1a1- Muc5b^Tg^, *Muc5b*^−/−^, and control mice were treated IT with bleomycin as in **d**. To induce similar levels of fibrosis while limiting survivor bias, SFTPC-Muc5b^Tg^ and controls received 2.0 U/kg bleomycin on day 0, followed by 1.0 U/kg on days 14 and 28. HP content increased in Scgb1a1-Muc5b^Tg^ and SFTPC-Muc5b^Tg^ but decreased in *Muc5b*^−/−^ mice compared to wild type (+) controls for each strain. **g**–**i** Fibrillar collagen (magenta in **g**) was assessed in peripheral lung tissues by confocal/multi-photon fluorescence microscopy with SHG. **h**, **i** Fibrillar collagen volumes following bleomycin were increased per mouse **h** and showed heterogeneous distributions **i** in Tg mice compared to controls. Data in **i** were analyzed by *t-*test (*n* = 105 Scgb1a1-Muc5b^Tg^ and 105 Scgb1a1-Muc5b^+(wt)^ images, and *n* = 120 SFTPC-Muc5b^Tg^ and 120 Scgb1a1-Muc5b^+(wt)^ littermate images. Scale bars, 10 μm **a**, **b**, **c**, 250 μm **e**, and 100 μm **g**. In **f** and **h**, data are means ± sem, numbers in italics indicate *n* animals used per experiment, and *p*-values indicate differences determined by ANOVA with Holm-Sidak’s multiple comparisons test. In **i**, * indicates statistical significance (*p* < 10^−5^)

In human IPF lung tissue, we found that *MUC5B* is co-expressed with surfactant protein C in columnar epithelial cells lining honeycomb cysts (Fig. 1a) and in type 2 alveolar epithelia (Fig. 1b), indicating that cell types involved in lung fibrosis in the distal airspace also express MUC5B. Accordingly, to understand the effect of increased *Muc5b* expression, such as that associated with the *MUC5B* promoter variant, we generated two lines of C57BL/6 mice that overexpress full-length murine *Muc5b* genomic transgenes. Tg(Scgb1a1-Muc5b) overproduce Muc5b under the control of a mouse secretaglobin 1a1 promoter fragment^10^, and Tg(SFTPC-Muc5b) mice overproduce Muc5b under the control of a human surfactant protein C promoter fragment (Supplementary Fig. 1a,b). These non-targeted transgenic lines are referred to as Scgb1a1-Muc5b^Tg^ and SFTPC*-*Muc5b^Tg^, and constitutively overexpress Muc5b in specific lung locations in addition to endogenous Muc5b (Supplementary Fig. 1c-f). Notably, Scgb1a1-Muc5b^Tg^ mice produce Muc5b throughout the conducting airways, whereas SFTPC-Muc5b^Tg^ mice produce Muc5b in the distal airways and alveoli (Fig. 1c and Supplementary Fig. 1c). *Muc5b* gene-deficient mice on a congenic C57BL/6J background (*Muc5b*^−/−^) were included to assess the effect of the absence of Muc5b^10^.

Collectively, these mouse lines allowed for robust gain-of-function and loss-of-function analyses of the effects of Muc5b production, and its effect in mouse models of pulmonary fibrosis.

Initially, mice were challenged with three intratracheal (IT) doses of bleomycin (2.5 U/kg, 1.5 U/kg, and 1.5 U/kg) over 7 weeks to strongly simulate the temporal heterogeneity and progressive nature of human IPF^11^. SFTPC-Muc5b^Tg^ mice express substantially higher concentrations of Muc5b than Scgb1a1-Muc5b^Tg^ mice (Supplementary Fig. 1d-f). Furthermore, following challenge with bleomycin, SFTPC-Muc5b^Tg^ mice experience significantly reduced survival than Scgb1a1-Muc5b^Tg^, *Muc5b*^−/−^, and wild-type control mice for each strain (Fig. 1d). To minimize survivor bias, we used a lower dose of bleomycin (2.0 U/kg, 1.0 U/kg, and 1.0 U/kg) in SFTPC-Muc5b^Tg^ mice for subsequent studies.

Following bleomycin challenge, both Scgb1a1-Muc5b^Tg^ and SFTPC-Muc5b^Tg^ mice demonstrate substantial lung injury that was less prominent in *Muc5b*^−/−^ mice (Fig. 1e). Therefore, we assessed lung fibrosis biochemically by measuring hydroxyproline (HP) content, a marker of collagen deposition. Bleomycin challenged Scgb1a1-Muc5b^Tg^ and SFTPC-Muc5b^Tg^ mice have elevated amounts of lung HP compared to their transgene negative littermate controls (Fig. 1f). In contrast, mice lacking Muc5b (*Muc5b*^−/−^) demonstrate decreased HP compared to wild-type littermates (Fig. 1f). This difference in collagen was quantified using confocal/multiphoton-excitation fluorescence microscopy with second harmonic generation (SHG, Fig. 1g). Quantification of SHG images demonstrates that Scgb1a1-Muc5b^Tg^ and SFTPC-Muc5b^Tg^ mice have significantly more collagen than non-challenged and transgene-negative littermate controls following challenge with bleomycin (Fig. 1h, i). Thus, *Muc5b* expression correlates with fibrosis detected biochemically and histologically, suggesting that the presence of excess Muc5b enhances the fibrotic response to bleomycin and that absence of Muc5b diminishes this response.

### Effects of Muc5b in distal airspaces on MCC

**Fig. 2:**
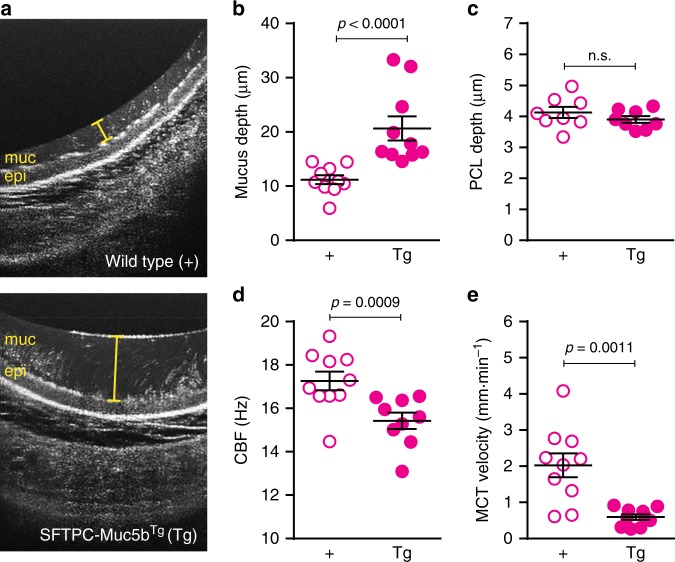
Muc5b overexpression in SFTPC-Muc5bTg mice is associated with impaired mucociliary transport. **a** Representative μOCT images of excised SFTPC-Muc5bTg mice trachea in comparison to trachea of wild type (+) littermate controls. **b**–**e** Quantitative metrics from image analysis reveal increased mucus layer depth **b** without significant alteration of periciliary layer (PCL) depth **c** in Tg mice compared to + controls. Functional analysis demonstrated reduced ciliary beat frequency **d** and diminished mucociliary transport rates **e** in Muc5b-overexpressing mice. Data in **b**–**e** are means ± sem and were analyzed by Mann–Whitney test

To explore a mechanism involved in the Muc5b-associated fibrotic response to bleomycin, we considered the effect of enhanced expression of Muc5b on MCC^12,13^. Examination of the tracheal mucus layer by micro optical coherence tomography (μOCT)^14–16^ shows distinct differences in mucociliary transport apparatus between SFTPC-Muc5b^Tg^ and transgene negative littermates (Fig. 2a and Supplementary videos 1 and 2). Quantitatively, the mucus depth is significantly greater (Fig. 2b) in SFTPC-Muc5b^Tg^ mice compared to transgene-negative littermate control mice, but this has a minimal effect on the periciliary layer depth (Fig. 2c), suggesting that osmotic forces transmitted by increased mucus concentration are not substantial^12^. However, both ciliary beat frequency (Fig. 2d) and mucociliary transport rate (Fig. 2e) are disrupted in SFTPC-Muc5b^Tg^ mice. Mucin overexpression in Scgb1a1-Muc5b^Tg^ mice induces similar μOCT results (Supplementary Fig. 2, Supplementary videos 3 and 4), although both the mucus layer increase vs. respective littermate controls (7.0 in Scgb1a1-Muc5b^Tg^ vs. 9.5 in SFTPC-Muc5b^Tg^ mice) and the relative mucociliary transport decrement (49.5% and 70.6%, respectively) are less pronounced in this model, consistent with bleomycin sensitivity (Fig. 1d–i).

### Effects of mucolytic treatment on MCC and fibrosis

**Fig. 3:**
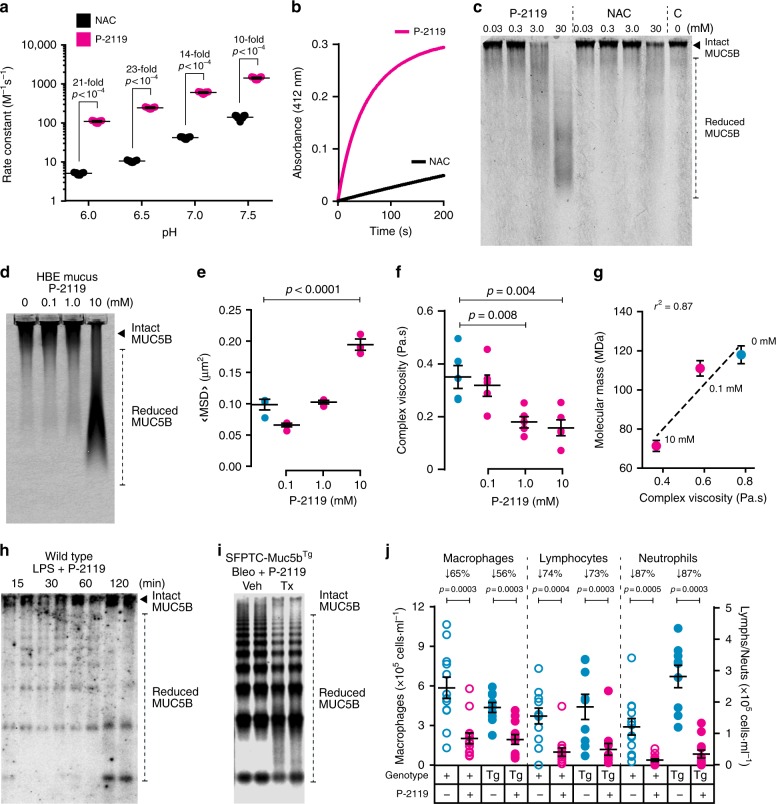
P-2119 effectively cleaves mucus in vitro and in vivo, enhancing the acute clearance of inflammatory cells. **a**, **b** P-2119 hydrolyzed DTNB disulfide bonds more quickly than n-acetylcysteine (NAC) **a**, and at pH 6 P-2119 cleaved more bonds than NAC **b**. **c** In human saliva, P-2119 reduced MUC5B in salivary mucus at lower concentrations than NAC. **d**–**g** In concentrated normal human bronchial epithelial cell culture mucus (5% solids), P-2119 reduced MUC5B at a potency similar to that seen in saliva in **c**. Reduction of MUC5B by P-2119 dose dependently lowered mucus viscosity as demonstrated by enhanced mean square displacement (MSD) of fluorescent microspheres **e**, and as shown by improved macrorheological complex viscosity **f** that was strongly correlated with reduced molecular mass **g**. Data in **e** represent 900 technical and three biological replicates; data in **f** represent three technical and two biological replicates. Cyan symbols, vehicle; magenta symbols, P-2119. **h**–**j** In vivo effects of aerosolized P-2119 (68–135 mM for 60 min). **h** Wild-type mice challenged with LPS (20 μg, IT) 48 h prior to P-2119 aerosol. P-2119 decreased Muc5b mass detected by immunoblot of lung lavage fluid over a 120 min period. **i** SFTPC-Muc5b^Tg^ mice were challenged with bleomycin (2.5 U/kg, IT) 7 d prior to P-2119 treatment (Tx). P-2119 caused Muc5b reduction detected by immunoblot 120 min post initiation of P-2119 aerosol. **j** The effect of P-2119-induced mucolysis on MCC was assessed by quantifying the acute elimination of leukocytes in lung lavage fluid obtained from bleomycin treated SFTPC-Muc5b^Tg^ mice (*n* = 9 vehicle and 12 P-2119 treated) and wild type (+) controls (*n* = 12 vehicle and 13 P-2119 treated). Total cells were significantly lower in P-2119 treated mice compared to vehicle treated animals, reflecting decreases in all leukocyte subtypes in bleomycin-injured lungs. Data in **a**, **e**–**g**, and **j** are means ± sem. Data in **a** were analyzed between P-2119 and NAC treated groups using *t*-tests. Data in **e**–**g** were analyzed statistically on biological replicates: ANOVA of results between 0 mM vehicle and 0.1–10 mM P-2119 treatment groups **e**, **f** and linear regression of complex viscosity vs mass **g**

The increase in fibrosis and decrease in mucociliary transport associated with elevated Muc5b levels led us to investigate whether therapeutic agents predicted to accelerate mucus clearance would be effective in improving outcomes in the SFTPC-Muc5b^Tg^ mouse model of lung fibrosis. Therefore, we tested the effects of a mucolytic compound, P-2119^17^ (Parion Sciences, Durham, NC), that is designed to hydrolyze disulfide bonds more efficiently than existing reducing agents, e.g. N-acetylcysteine (NAC). P-2119 yields faster disulfide bond reduction and at lower concentrations than NAC in assays utilizing a model disulfide bond substrates (5,5′-dithiobis-2-nitrobenzoic acid (Fig. 3a, b) and MUC5B purified from human saliva (Fig. 3c). Additionally, in concentrated human airway mucus the ability of P-2119 to reduce MUC5B (Fig. 3d) causes improvements in viscosity and elasticity that are consistent with mucus alterations predicted to facilitate clearance (Fig. 3e, f and Supplementary Fig. 3) and are directly related to decreased MUC5B molecular mass determined by multi-angle laser light scattering spectroscopy (Fig. 3g). With these studies demonstrating the effectiveness of a mucolytic agent in vitro, we next sought to assess the effectiveness of P-2119 in vivo. In WT C57BL/6J mice challenged with LPS to induce Muc5b expression and acute lung injury, P-2119 inhalation effectively depolymerizes Muc5b, and the mucolytic effect of P-2119 persists for 120 min (Fig. 3h). Having established dose and time course parameters in WT mice, we next tested the effects of P-2119 in SFTPC-Muc5b^Tg^ mice treated with a single IT dose of bleomycin. In these animals, P-2119 treatment also results in smaller mucin polymers detected in lung lavage fluid (Fig. 3i). Mucolytic treatment results in acute clearance of inflammatory cells from the lungs, which is demonstrated by a significant and rapid diminishment in lung lavage leukocyte numbers (Fig. 3j and Supplementary Fig. 4) concurrent with Muc5b depolymerization. In aggregate, these results suggest that P-2119, a mucolytic agent, may favorably influence mucus properties in the airspace by improving MCC, consequently minimizing fibrosis following bleomycin-induced lung injury in the context of Muc5b overproduction.

**Fig. 4:**
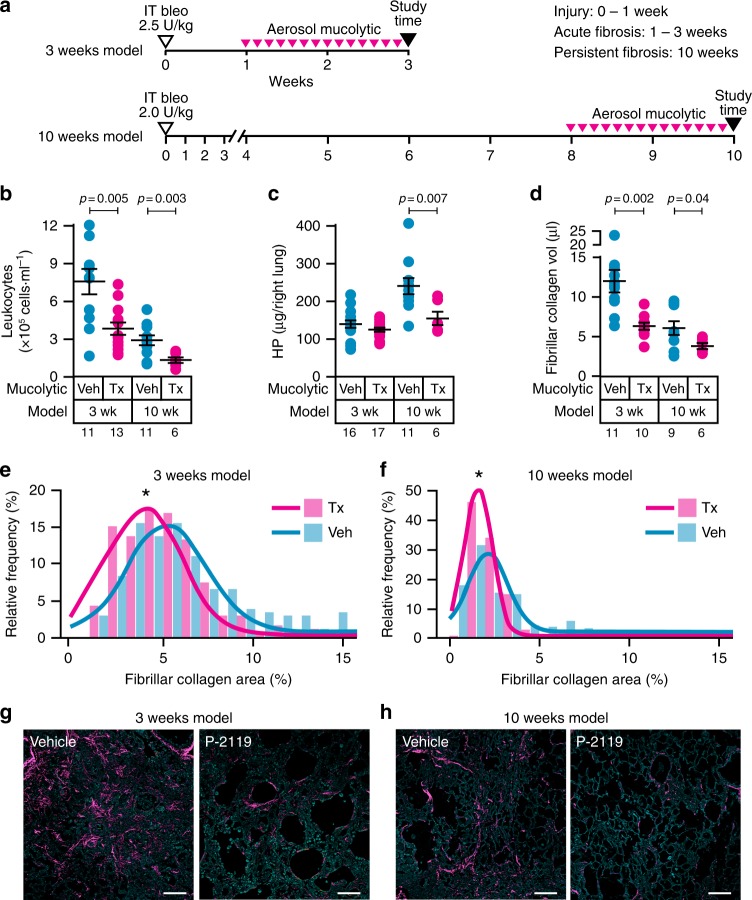
P-2119 treatment results in reduced collagen deposition in the setting of excess Muc5b. **a** SFTPC-Muc5bTg mice were subjected to bleomycin challenge with a 2 wk P-2119 intervention protocol, with daily P-2119 treatments (Tx) or saline vehicle (Veh) exposures starting 7 or 56 d post bleomycin challenge and ending 24 h prior to tissue harvest. **b**–**h** Injury and fibrosis induced in bleomycin-treated mice exposed to saline vehicle were decreased in P-2119 treated animals. Compared to vehicle treated animals, P-2119 treated mice had significantly fewer leukocytes in lung lavage **b**, less hydroxyproline in lung homogenates at 10 wks **c**, and decreased peripheral lung fibrillar collagen **d**–**h**. In **b**–**d**, data are means ± sem, numbers in italics indicate *n* animals used per experiment, and *p*-values indicate differences determined by *t*-test with Welch’s correction for unequal variances. In **e** and **f**, * indicates statistical significance by *t*-test (*p* < 0.00001). Histograms in **e** are from 15 Veh mice (225 images) and from 12 Tx mice (165 images); histograms in **f** are from 9 Veh mice (180 images) and from 6 Tx mice (120 images). Images in **g** and **h** show greater amounts of fibrillar collagen (red) in Veh vs. Tx groups. Scale bars, 100 μm **g**, **h**

To pursue this further, we focused on the SFTPC-Muc5b^Tg^ mice that produced the highest concentrations of Muc5b (see Supplementary Fig. 1d,f), were most responsive to bleomycin (Fig. 1), and had more pronounced changes in mucociliary transport (see Fig. 2 and Supplementary Fig. 2). SFTPC-Muc5b^Tg^ mice were challenged with 2.5 U/kg of bleomycin IT, were allowed to respond to bleomycin for 1 or 8 weeks, and were then exposed to 2 weeks of aerosolized P-2119. Hence, mice were observed at 3 or 10 week endpoints post-bleomycin (Fig. 4a). This comprehensive approach allowed us to test the ability of P-2119 to treat both short and long-term fibrotic lung responses induced by bleomycin, whereas leaving the effects of bleomycin on lung injury intact^18^. Treatment with P-2119 reduces the severity of chronic inflammation (Fig. 4b, Supplementary Fig. 5), as well as mortality associated with bleomycin-induced lung injury (Supplementary Fig. 6). These protective effects of P-2119 on lung injury are not associated with changes in redox balance (Supplementary Fig. 7) or inflammatory cytokine/chemokine expression (Supplementary Table 1).

To assess the anti-fibrotic effects of mucolytic treatment, collagen levels in mouse lung tissues were quantified using biochemical and histological assays. Whereas, we do not detect a difference in HP content in lung homogenates between P-2119 and saline in short term studies (3 weeks after bleomycin), persistent HP increases are observed in SFTPC-Muc5b^Tg^ mice over a longer period (10 weeks) and these changes are significantly reduced by P-2119 treatment (Fig. 4c). Importantly, histologically detectable fibrillar collagen detected by SHG imaging decreases in the lungs of SFTPC-Muc5b^Tg^ mice treated with P-2119, and this is observed in both the 3 week and 10 week model of bleomycin-induced lung fibrosis (Fig. 4d–h). Strikingly, in the absence of P-2119 treatment, HP values increase in SFTPC-Muc5b^Tg^ mice during prolonged responses to bleomycin (Fig. 4c). However, the concentration of fibrillar collagen decreases over time (Fig. 4d). This discrepancy could reflect differences in the types of collagen detected since unlike SHG, HP does not distinguish fibrillar vs. non-fibrillar types of collagen. Also, SHG image analyses did not include central airways and vasculature, but were instead conducted on peripheral lung tissues (the location of pulmonary fibrosis in humans). Importantly though, for both HP and SHG analyses, P-2119 significantly reduces acute and protracted lung fibrosis. Taken together, these findings highlight the importance of short and long-term models of lung fibrosis and comprehensive assessment of outcomes, including secondary endpoints, such as survival, along with the critical primary endpoint of fibrosis in parenchymal lung tissues.

## Discussion

Mucociliary dysfunction is an emerging paradigm in lung diseases^19,20^. Previously considered a characteristic specific to obstructive diseases such as asthma and chronic obstructive pulmonary disease, and genetic diseases such as primary ciliary dyskinesia and cystic fibrosis, the importance of mucins, mucus, and mucociliary interactions has surfaced in diseases of the lung periphery, such as adenocarcinoma and IPF^4,5^. Unlike obstructive diseases, which involve central conducting airways and rapid defense against ubiquitous environmental challenges, mucus dysfunction in the lung periphery appears to involve injury-repair processes predominantly^5,10,21^. In this vein, infection, mucous metaplasia, and bronchiectasis that occur with mucociliary dysfunction in conducting airways contrast with pathologic changes, such as bronchioloalveolar epithelial and mesenchymal remodeling or proliferation that occur with mucin overexpression in the lung periphery^21–26^.

Our results demonstrate that elevated concentrations of Muc5b in the distal lung are directly related to the fibroproliferative response to bleomycin in mice, and MCC dysfunction might play an important role in this response. Previous findings in knockout mice showed MCC failure in the absence of Muc5b^10^, so dysfunctional MCC due to high concentrations of Muc5b may seem counterintuitive. However, the distribution of mucous and ciliated cells, the anatomical location of *MUC5B/Muc5b* expression, and the homeostatic control of mucus hydration are factors that coordinately affect mucus viscoelasticity and transport^5^. Accordingly, ectopic overproduction of Muc5b in the lung periphery in SFTPC-Muc5b^Tg^ mice (see Fig. 1c and Supplementary Fig. 1c) produces more severely defective mucociliary transport than what is observed in Scgb1a1-Muc5b^Tg^ mice (see Fig. 2e and Supplementary Fig. 2e), where Muc5b overproduced by club cells in airways where it is also normally expressed^27–29^. These findings suggest that overexpression of *MUC5B* in distal airspaces, which is known to occur in IPF^4,9^, disrupts the equilibrium necessary to sustain effective mucociliary transport^13,30^ thereby impairing mucus function^12,26,31^.

One potential consequence of mucociliary dysfunction is retention of inhaled substances (air pollutants, cigarette smoke, microorganisms, etc.) and endogenous inflammatory debris that, over time, results in temporally and spatially distinct areas of microscopic scaring and progressive fibroproliferation in the lung leading to the development of IPF^1^. Alternatively, reduced clearance or enhanced viscosity of MUC5B may initiate a reactive or regenerative fibrotic response localized to the bronchoalveolar region of the lung that eventually leads to the development of IPF. As we observed that MUC5B is co-expressed with surfactant protein C in type 2 alveolar epithelia and cells lining honeycomb cysts in human IPF (Fig. 1a, b), we postulate that the cells involved in MUC5B overexpression are involved in the lung remodeling that is characteristic of IPF.

Whereas there is no true equivalent animal model of IPF, intratracheal bleomycin challenge is a widely used model of lung fibrosis in mice. Nonetheless, both bleomycin challenge as a trigger and the mouse as a species have strengths and weaknesses^32^. Bleomycin causes an acute increase in collagen production that can resolve over time, but bleomycin can induce prolonged lung fibrosis under some conditions, including with repeated dosing^11,33^ as we have done here (Fig. 1c–i). In addition, mouse lungs are anatomically different from humans. Mice lack respiratory bronchioles and demonstrate little or no mucin expression in the lung periphery^27–29^. By using SFPTC-Muc5b^Tg^ mice, we help to significantly narrow this species gap. Importantly, SFTPC-Muc5bTg mice developed prolonged fibrosis in a single bleomycin challenge setting (Fig. 4). Thus, even in an acute injury setting that differs from the chronic development of disease in humans, this Muc5b-overexpressing model provides useful insight in an IPF-related setting. Other aspects such as microscopic honeycombing and proliferative repair vs. remodeling need to be explored^11,33^. The last has been implicated with much of the focus on expansion of fibroblasts and alveolar remodeling suggestive of a process of active disease progression. Our findings support a role for mucociliary dysfunction in active disease, and they support a long-term goal in the field of identifying ways to slow or reverse the development of IPF at early and/or preclinical stages.

The potential role for mucociliary dysfunction as a driver of IPF pathology is supported by unique gene expression signatures in IPF. In patients with IPF, the coordinated overexpression of *MUC5B* and cilium genes is associated with microscopic honeycombing, a pathognomonic feature of IPF^34^. Furthermore, cilium and *MUC5B* gene expression are associated with the concentration of *MMP7*, a metalloproteinase gene that is a known biomarker in IPF^35^ and is also known to amplify airway ciliated cell differentiation^36^. Adequate MCC requires a balance between the concentrations of water, ions, and macromolecules in the mucus gel. Excessive production of MUC5B presents a challenge to proper mucus hydration and cilia function^12^. In equilibrium, airway surface liquid homeostasis maintains a low-friction state within the periciliary layer (PCL). In diseases such as cystic fibrosis, a strong transcellular osmotic gradient causes hyperabsorption of ions and water by airway epithelial cells, generating mucus dehydration, reduced cilia motility, and in extreme cases cilia collapse. This phenomenon can also be caused by the presence of high concentrations of mucins on the apical surfaces of airways, generating similar dehydrating forces as observed in CF; excessive mucin could result in an osmotic gradient favoring water movement out of the PCL and towards airway lumen^12,37^. In both cases, the loss of a grafted gel-on-brush confirmation results in excessive mucus aggregation and impaired MCC. Finally, when mucin expression is uncoupled with CFTR-dependent anion secretion, abnormally viscous mucus could ensue beyond the effects of airway dehydration, precipitating abnormal host defense^15^. Whereas the findings here do not support PCL depletion as a mechanism for mucociliary dysfunction in Muc5b-overproducing mice at baseline (see Fig. 2c and Supplementary Fig. 2c), it is possible that PCL depletion may be detectable in injured mice. Additional studies will identify how these biophysical properties are regulated and alter mucociliary function, as well as the extent to which genetic factors such as the *MUC5B* promoter variant rs35705950 impact airway epithelial cell biology.

The relationship between MUC5B overproduction and IPF is complex. Although Muc5b transgenic mice do not appear to spontaneously develop pulmonary fibrosis, they are more responsive to bleomycin (Fig. 1f–i). Likewise, the human gain-of-function *MUC5B* promoter variant that causes overexpression of *MUC5B* in the distal airspace also does not appear to be sufficient to cause pulmonary fibrosis. Although ~20% of non-Hispanic Whites have at least one copy of the *MUC5B* promoter variant^4^, IPF is a rare disease occurring in less than 0.1% of the population^1^. Despite the caveat that IPF is likely underdiagnosed^38–40^, it remains clear that the *MUC5B* promoter variant represents a low penetrance allele. It is thus likely that other gene variants and/or environmental exposures interact with the *MUC5B* promoter polymorphism to cause IPF in individuals with this disease-associated genetic variant. These observations lead us to postulate that the etiology of IPF will best be understood by identifying the genes, transcripts, and environmental exposures that interact with *MUC5B* and contribute to the development of IPF. While future studies in large populations of patients with IPF may reveal the critical biological mechanisms that interact with the gain-of-function MUC5B promoter variant to cause IPF, our Muc5b transgenic mice may prove critically important in identifying the genes and/or environmental exposures that contribute to this complex disease.

In a broader context, these findings suggest that by identifying those at risk, patients could be diagnosed prior to the development of permanent and extensive lung parenchymal scarring. Genetic risk factors, particularly the *MUC5B* promoter variant^4^, have been shown to identify individuals with preclinical interstitial changes on chest CT scan^38–40^ that progress to clinical significance and are associated with reduced survival^39^. Given the irreversible nature of IPF, even approved treatments (pirfenidone^2^ and nintedanib^3^) only modestly slow progression and have not been shown to alter the median 3–5 year survival. Patients with preclinical stages of pulmonary fibrosis may be ideal candidates for early intervention focused on avoiding the development of irreversible lung remodeling. Our findings suggest that targeting MUC5B in the terminal airways of patients with preclinical stages of interstitial lung disease represents a rational strategy to prevent the progression of preclinical pulmonary fibrosis.

## Methods

### Human lung specimens

Lung specimens from patients with idiopathic pulmonary fibrosis (IPF) and unaffected controls were obtained from the NHLBI Lung Tissue Research Consortium (LTRC; https://ltrcpublic.com/). All protocols were performed in compliance with all relevant ethical regulations approved by local Institutional Review Boards, and individuals gave written informed consent to participate.

### Mouse husbandry

Studies using animals complied with all relevant ethical regulations. Mice were housed in accordance with the Institutional Animal Care and Use Committee of the University of Colorado and University of Alabama at Birmingham and kept in specific-pathogen free housing areas that were monitored by institutional animal care staff. Mice were maintained on a 12 h light/dark cycle and fed ad libitum a normal diet of water and irradiated chow (Harlan Teklad). Mice were assigned non-descriptive numbers at weaning by attachment of an ear tag. Moribund mice were identified by observing changes in body weight and behavior. In accordance with the veterinary care procedures at University of Colorado, a loss of 15% of body weight without recovery, hunching, fur ruffling and lethargy were used as criteria for determining moribundity. Animals were killed by intraperitoneal injection of sodium pentobarbital followed by exsanguination.

### Genetically engineered mice

Muc5b^+/−^ mice were generated previously^10^, and heterozygous males and females were bred to obtain male Muc5b^−/−^ and Muc5b^+/+^ littermates for experiments. Scgb1a1-Muc5b^Tg^ mice were generated previously^1^ and were maintained by continuous hemizygous outcrosses with C57BL/6J male mice purchased from the Jackson Laboratory. SFTPC-Muc5b^Tg^ mice were created by insertion of the full-length 34-kb genomic coding region of the mouse *Muc5b* gene into a transgenic targeting cassette containing 3.7 kb of the human *SFTPC* gene 5′-flanking region and an IRES-mCherry reporter. Founders were generated by injecting the targeting vector into C57BL/6N pronuclei at the National Jewish Health transgenic mouse core. After confirmation of Muc5b overexpression by western blot, SFTPC-Muc5b^Tg^ mice were maintained by continuous hemizygous outcrosses with C57BL/6J male mice purchased from the Jackson Laboratory.

### Bleomycin exposure

All mice began bleomycin treatment between 8 and 12 weeks of age, and only male mice were used due to their known sensitivity to bleomycin^41,42^. Mice were anesthetized with inhalational isoflurane (MWI Veterinary Supply Company, Boise, ID) and tracheas were directly visualized with a rodent laryngoscope (Penn Century, Wyndmoor, PA). A 22 g gavage needle was used to instill 50 µl of saline or bleomycin solution (APP Pharmaceuticals, Schaumburg, IL) directly into the trachea. Studies were conducted following repeated bleomycin challenges (Fig. 1) or following a single bleomycin challenge (Figs. 3, 4 and Supplementary Fig. 4). For repeated bleomycin challenge studies shown in Fig. 1d, e, animals received 2.5 U/kg bleomycin in d 0, followed by 1.5 U/kg on d 14 and d 28. For studies shown in Fig. 1f–i, SFTPC-Muc5b^Tg^ mice received 2.0 U/kg on d 0, followed by 1.0 U/kg on d 14 and d 28. All repeat challenge animals were collected on d 49. For single bleomycin challenge studies, SFTPC-Muc5b^Tg^, mice received 2.5 U/kg on d 0 and were studied on d 7 (Fig. 3), d 21 (Fig. 4), or d 70 (Fig. 4).

### Bronchoalveolar lavage

Immediately following euthanasia, mouse tracheas were cannulated and the lungs lavaged three times with 0.5 ml of PBS containing 0.6 mM EDTA. Cells were counted using a hemacytometer and spun on to slides using a Cytospin 4 (Thermo Fisher Scientific, Waltham, MA). The slides were stained with the Hema 3 kit (Thermo Fisher) and used for differential counts of macrophages, lymphocytes, and neutrophils. Remaining lavage fluid was divided into two aliquots. One portion was centrifuged at 300×*g* for 10 min at 4 °C for subsequent studies of cytokines and other soluble factors, and the other portion was stored without centrifugation to preserve high molecular weight mucins that sediment at low centrifugation speeds. Both were frozen on dry ice and stored at −80 °C.

### Hydroxyproline

For repeat bleomycin studies, the entire right lung was removed, added to 550 μl PBS and homogenized using Lysing Matrix D and a FastPrep-24 bead beater (MP Biomedicals, Santa Ana, CA). Samples were then snap frozen and stored at −80 °C. Thawed lung homogenates were hydrolyzed overnight with a 1:1 volume of 12N HCl at 100 °C. Afterwards, 5 μl samples of hydrolyzed lung and hydroxyproline standards were plated in duplicate in 96-well plates and incubated for 20 min in 100 μl of 0.06 M chloramine T in citrate-acetate buffer, pH 6. Ehrlich’s solution (100 μl; 1.2 M dimethylaminobenzaldehyde in 22% perchloric acid-*n*-propanol) was then added to each sample. After a 20-min incubation at 65 °C, plates were analyzed in an Synergy H1 plate reader (Biotek, Winooski, VT) at 550 nm for colorimetric analysis. Concentrations of each sample were determined by interpolation along a standard curve.

For P-2119 studies^17^, the right upper, lower, and accessory lobes were homogenized in 500 μl PBS and then processed as above.

### Histochemistry and immunohistochemistry

At harvest, the left lung was inflated with 4% paraformaldehyde (PFA) at a pressure of 20 cm H_2_O for 5 min. The lung was then removed and placed in fresh 4% PFA overnight for fixation. Left lungs were cut into uniform slices, and volumes were recorded using Cavalieri imaging calculations. Lungs were then embedded in paraffin, cut into 5 µm sections, and collected on positively charged glass slides. For hematoxylin and eosin staining, tissues were stained using standard reagents at the University of Colorado Cancer Center Pathology Core.

For immunohistochemistry, tissues were heated in citrate buffer for antigen retrieval (20 min boiling), and tissues were then incubated overnight in goat primary antisera against Muc5b (1:1000, Everest Biotech, Upper Heyford, UK) or with rabbit primary anti-mouse Muc5b antisera (1:20,000)^10^. Secondary anti-goat antibody diluted 1:1000 tagged with AlexaFluor488 (Thermo Fisher) or ImmPRESS anti-rabbit conjugated with horseradish peroxidase (Vector Laboratories) were applied for 1 h at room temperature. Immunofluorescence slides were coverslipped with VECTASHIELD Hardest mounting medium with DAPI (Vector). DAB stained slides were counterstained with hematoxylin and permanently mounted. Samples were visualized using an Olympus BX63 microscope (Olympus, Tokyo, Japan).

### In situ hybridization

RNAScope detection was used to perform in situ hybridization according the manufacturer’s protocol (Advanced Cell Diagnostics, Hayward, CA). Briefly, formalin-fixed paraffin embedded human lungs were cut into 5 µm thick tissue sections. Slides were deparaffinized in xylene, followed by rehydration in a series of ethanol/water washes. Following citrate buffer (Advanced Cell Diagnostics) antigen retrieval, slides were rinsed in deionized water, and immediately treated with protease (Advanced Cell Diagnostics) at 40 °C for 30 min in a HybEZ hybridization oven (Advanced Cell Diagnostics). Probes directed against *MUC5B* and *SFTPC* mRNA and control probes were applied at 40 °C in the following order: target probes, preamplifier, amplifier; and label probe for 15 min. After each hybridization step, slides were washed two times at room temperature. Chromogenic detection was performed followed by counterstaining with hematoxylin (American MasterTech Scientific, Lodi, CA). Staining was visualized using an Olympus BX63 microscope using a ×60 oil immersion lens, z-stack imaging, extended focal image processing (Olympus, Tokyo, Japan).

### Second harmonic generation (SHG)

Autofluorescence and SHG signals were acquired using Zeiss 780 laser-scanning confocal/multiphoton-excitation fluorescence microscope with a 34-channel GaAsP QUASAR detection unit and non-descanned detectors for two-photon fluorescence (Zeiss, Thornwood, NY). The imaging settings were initially defined empirically to maximize the signal-to-noise ratio and to avoid saturation; and they were kept constant for all measurements for comparative imaging and results. Seven percent of a two-photon Chameleon laser tuned to 800 nm was used for excitation, and emission signals corresponding to the autofluorescence and SHG signals were detected simultaneously through non-descanned detectors. Image processing was performed using Zeiss ZEN 2012 software. Fifteen images were obtained for each of the lung using standardized uniform random sampling^43^. The series of images were analyzed in Image J software. Percentage of area covered by fibrillar collagens was quantified for each slide, and collagen fractions were normalized to lung volumes. Histograms for cumulative data for each group was created using GraphPad Prism (GraphPad, La Jolla, CA).

### Micro-optical coherence tomography

Adult SFTPC-Muc5b^Tg^ and Scgb1a1-Muc5b^Tg^ mice at 8–12 weeks old and their age-matched littermate were killed using ketamine and xylazine. Tracheas were removed, separated from the distal airways and lung tissue, and placed in HEPES-buffered DMEM. Tracheal tissue was then dissected along the transverse axis to expose the luminal epithelial cell surface and incubated for 30 min under physiologic conditions (37 °C, 5% CO_2_, 100% humidity) to allow the epithelial surface to equilibrate.

Functional assessments of the tracheal tissue explants were performed using µOCT, with acquisition speed set at 20,480 Hz line rate to yield 100 frames per second at 256 lines per frame. Images were recorded in 8–10 ROI per trachea by an imaging specialist blinded to genotype. Images were recorded at randomly chosen intervals on the mucosal surface with the optical beam scanned along the longitudinal direction^16,44^.

Several metrics were simultaneously quantified from µOCT-recorded images using Image J and Matlab. Airway surface liquid (ASL) and Periciliary layer (PCL) depths were measured directly^44^. Mucociliary transport rate was calculated based on the slope of mucus particulates in the mucus over several frames in the ASL region up to 50 µm above the epithelial cell surface. CBF was determined using Fourier transform analysis of the reflectance of beating cilia. Imaging analysis was performed in a blinded fashion with respect to genotype.

### In vitro mucolytic testing

The rate of thiol-disulfide exchange was assayed for NAC and P-2119 utilizing Ellman’s reagent (5,5′-dithiobis-2-nitrobenzoic acid or DTNB) and methods adapted by Han and Han^45^. Briefly, NAC or P-2119 was added to a final concentration of 22.5 µM in an excess of DTNB (45 µM) and formation of the fluorescent DTNB cleavage product (2-nitro-5-thiobenzoate) was monitored over time at 412 nm by a spectrophotometer.

MUC5B was analyzed using whole saliva or mucus purified from primary human bronchial epithelial cell cultures and concentrated to 5% solids (w/v)^46^. Briefly, mucus samples were treated with reducing agents NAC or P-2119 (0.03–30 mM) for 1 h at room temperature. Mucin polymer reduction was assessed by agarose-gel western blot (see Supplementary Fig. 8 for uncropped scans). The mucus samples were alkylated with N-ethylmaleamide (100 mM final concentration) and mucins were separated by 1% (w/v) agarose-gel electrophoresis, vacuum-blotted onto a PVDF membrane, and detected by western blotting with MUC5B antibodies^47^.

### Mucus microrheology

Microbead rheology was performed by tracking the thermally driven motion of embedded 1 µm diameter carboxylated microspheres (FluoSpheres, Fischer Scientific)^37,48,49^. Briefly, microspheres were added to mucus and allowed to mix while rotating overnight at 4 °C. After reduction, fifteen 30 s movies were collect at 60 frames per second on a Nikon Eclipse TE 2000 microscope at ×40 with a Flea 3 camera (FLIR Machine Vision, Richmond BC, Canada). Particle trajectories were subsequently tracked using TrackPy (v2.4, 10.5281/zenodo.12255). The track positions were corrected in Matlab (The MathWorks, Natick, MA) to account for linear drift and mean squared displacement (MSD) was calculated for each bead according to Equation 1

1 $$\begin{array}{*{20}{c}} {{\mathrm{\Delta }}r^2\left( \tau \right) = \frac{1}{{N - \tau }}\mathop {\sum }\limits_{i = 1}^{N - \tau } \left( {x\left( {t_i + \tau } \right) - x\left( {t_i} \right)} \right)^2 \,+\, \left( {y\left( {t_i + \tau } \right) - y\left( {t_i} \right)} \right)^2\# } \end{array},$$

where *N* = 1800 total frames and *τ* is the time-lag.

### Mucus macrorheology

Mucus was collected from human bronchial epithelium (HBE) cell culture models^48,50,51^ and prepared to 5% solids to mimic the concentration seen in obstructive airways disease. Mucus was treated with concentrations of compound from 0.1 mM to 10 mM and allowed to incubate for 1 h at 37 °C. The rheological properties are determined by analyzing the linear regime of a fixed frequency stress sweep^50,51^. All assays were performed on a Discovery Hybrid 3 rheometer with 20 mm diameter 10 cone with solvent trap (TA Instruments, New Castle Delaware). Data were analyzed using custom Matlab scripts (MathWorks). All treatments we performed by mixing P-2119 in PBS at pH 7 to 100-fold higher than the desired final concentration, and 10 µl of compound was added to 90 µl of HBE mucus and incubated at 37 °C for 1 h. All assays were performed at 23 °C to prevent evaporation.

### Multi-angle laser light scattering spectrometry

The molecular weight determination is performed on a aliquot of the same mucus that was used for rheology. Ten microliters of mucus is added to 40 μl of 6M guanidinium HCl. Samples were then further diluted 1:20 into light scattering buffer for high-pressure liquid chromatography (HPLC) on a CL2B column to separate large molecular weight mucins from other small proteins. HPLC was run in combination with refractometry (TRex, Wyatt) and multi-angle laser light scattering (MALLS, DAWN Heleos II, Wyatt Technologies) to determine molecular weight (shown) and mucin concentration (unchanged by reduction).

### Aerosol exposure

Solutions were nebulized with an Aeroneb nebulizer. Aerosolized P-2119^17^ was delivered to a 24-port nose-only inhalation chamber (In-Tox Products, Inc., Albuquerque, NM) operated at ~10 l/min. Exposure monitoring was conducted by collection of air samples from the test atmosphere on to 47 mm Teflon membrane filters (TEFLO, Pall-Gelman) that were weighed before and after sample collection. The aerosol was withdrawn directly from the exposure chamber atmosphere with a flow rate of ~1 liter/minute. Particle size was ~ 1 μm with a geometric standard deviation of ~2.0.

### In vivo mucolytic testing

To test mucolytic activity, mice were tested under two conditions. First, wild-type C57BL/6J mice were challenged IT with 20 μg of LPS (*E. coli* 055:B5) administered in a 50 μl volume of saline. Mice were then treated 2 d post LPS challenge with P-2119 (135 mM) or vehicle (normal saline) for 60 min. Mice were studied 15, 30, 60, and 120 min post P-2119 treatment (68 mM aerosol) to evaluate the potency and kinetics of mucolysis induced by P-2119. Second, a separate group of SFTPC-Muc5b^Tg^ mice was then analyzed following a single IT bleomycin challenge. Bleomycin treated mice were then treated with P-2119 (135 mM) or vehicle (normal saline) by aerosol for 60 min on d 7 post challenge, a peak point of bleomycin-induced inflammation. Mice were studied immediately after withdrawal from P-2119 or vehicle challenge or 1 h after P-2119 (60 and 120 min timepoints post initiation of treatments, respectively). Mucolysis was assessed in un-centrifuged lung lavage fluid that was treated with 1 M iodoacetamide to quench drug activity and alkylate thiols liberated by disulfide reduction. Western blotting was performed as described above for human MUC5B, in this case with rabbit-anti-mouse Muc5b antisera.

We further evaluated the functional consequences of mucolytic treatments mouse lungs. The same LPS (d 2), bleomycin (d 7), and mucolytic treatment groups described above for assessing mucolysis biochemically were used to assess mucolytic effects on MCC and mucus obstruction. To test MCC, we developed an acute endogenous clearance (AEC) measurement. Leukocyte numbers in lung lavage fluid were enumerated in P-2119 and vehicle control groups. AEC was determined by quantifying an acute decrease in leukocyte numbers in lavage fluid. In addition, to quantify mucus elimination, distal airspace Muc5b was examined immunohistochemically by fixing air-inflated lungs via immersion in methacarn^21,52^. Fractions of randomly selected ventral alveolar airspaces occupied by Muc5b were assessed by point counting^21^.

### Cytokine and chemokine assessment

Chemokines and cytokines were assessed using a 19-plex MSD Multi-spot Assay System in bronchoalveolar lavage fluid supernatant obtained from mice exposed to saline (untreated) or P-2119 treatment (treated). Data are presented in pg/ml of each analyte (Supplementary Table 1). Statistical analysis was performed using a Mann–Whitney test.

### Redox balance testing

Levels of glutathione (GSH) and oxidized glutathione (GSSG) were assess using a GSH-Glo™ Glutathione Assay (Promega, Madison, WI). The protocol (384-well format) was modified from the manufacturer’s protocol (96-well format, Promega V6911). In brief, 2.5 µl of unclarified lung lavage samples (in duplicate) were treated with 2.5 µl GSH-Glo reagent with or without TCEP (final concentration = 1 mM). Following a 30 min incubation at room temperature, luciferin detection reagent (5 µl) was added, and luminescence signals were recorded on a plate reader (PerkinElmer EnVision 2104, 700 nm, 0.5 s exposure) 15 min later. For reactive oxygen species (ROS), samples were tested using a ROS-Glo Assay (Promega). Lung lavage samples were prepared as described above for GSH-Glo assays. Following a 60 min incubation at room temperature, luciferin detection reagent (5 µl) was added, and luminescence signals were recorded on a plate reader (PerkinElmer EnVision 2104, 700 nm, 0.5 s exposure) 20 min later.

### Statistical analysis

Hydroxyproline, redox, inflammation, and gene expression data were analyzed by *t*-test or non-parametric analyses for non-normally distributed data, and parametric or non-parametric ANOVA’s for multiple comparisons (GraphPad, La Jolla, CA). SHG percentage area histograms were fitted with Gaussian model using a least *χ*^2^ fit model to determine numbers of peaks in each experimental group in IgorPro (WaveMetrics, Lake Oswego, OR) and each pair was analyzed using a *t*-test. Degrees of freedom were over 100 for each distribution, giving a rejection region of 3.09 to ∞ for critical values (t) at a 0.1% level of significance.

## Supplementary information

Supplementary Information

Description of Additional Supplementary Files

Supplementary Video 1

Supplementary Video 2

Supplementary Video 3

Supplementary Video 4

## Data Availability

The data that support the findings of this study are available from the authors on reasonable request, see author contributions for specific data sets.
