# Supplementary material for: Muc5b overexpression causes mucociliary dysfunction and enhances lung fibrosis in mice
Source: Nat Commun. 2018 Dec 18;9:5363. doi: 10.1038/s41467-018-07768-9 (PMC6299094; doi:10.1038/s41467-018-07768-9)
Supplement: Supplementary file 1 — Supplementary Information [file 41467_2018_7768_MOESM1_ESM.pdf]

Muc5b overexpression causes mucociliary dysfunction and enhances lung fibrosis in mice

Hancock et al.

Supplementary Information

Supplementary Figures 1-8

Supplementary Table 1

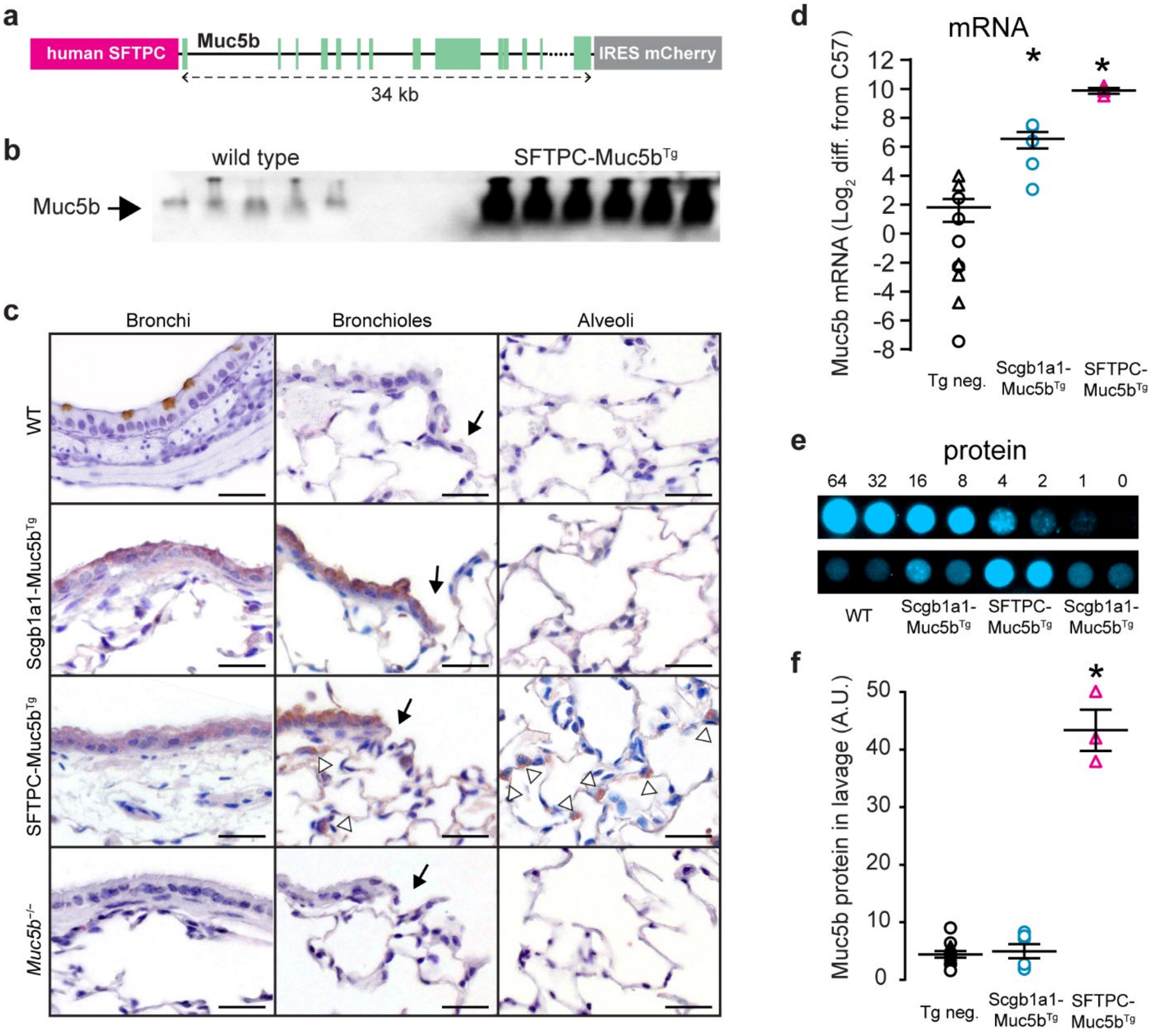

Supplementary Figure 1. Transgenic models for Muc5b overproduction. **(a)** A full length (34 kb) fragment of the mouse *Muc5b* gene was inserted into a transgenic targeting vector containing 3.7 kb of the human surfactant protein C (SFTPC) promoter and an internal ribosomal entry sequence (IRES)-monomeric Cherry (mCherry) red fluorescent protein. **(b)** Mice possessing this SFTPC-Muc5b transgene (SFTPC-Muc5b^{Tg} mice), overproduce Muc5b in the lungs. Western blot analysis of Muc5b extracted from lysates of SFTPC-Muc5b^{Tg} mice and wild type littermate control. **(c-f)** Comparison of Muc5b expression and localization in Muc5b mutant animals. **(c)** Immunohistochemistry performed using rabbit-anti-Muc5b antisera (1:20,000) shows Muc5b localization to bronchial airways in wt mice, with extension of Muc5b to distal bronchioles in Scgb1a1-Muc5b^{Tg} mice, and to distal bronchioles and type 2 alveolar epithelia in SFTPC-Muc5b^{Tg} mice. Muc5b immunolabeling was absent in *Muc5b*$^{-/-}$ lung tissues. Arrows, bronchoalveolar duct junctions. Arrowheads, Muc5b expressing type 2 cells. Scale bar, 50 µm. **(d)** Compared to wt (n = 12), Muc5b mRNA levels in lung lysates were 50-fold higher in Scgb1a1-Muc5b^{Tg} (n = 6) mice and 150-fold higher in SFTPC-Muc5b^{Tg} mice (n = 3). **(e,f)** Muc5b levels were assessed in lung lavage fluid by dot blot ELISA **(e)**. Compared to wt (n = 6) levels in lung lavage fluid **(f)**, Muc5b protein levels were not significantly increased in Scgb1a1-Muc5b^{Tg} mice (n = 6), and were >40-fold higher in SFTPC-Muc5bTg mice (n = 3). In **e,f**, circles depict Scgb1a1-Muc5bTg mice and their wild type littermates, and triangles depict SFTPC-Muc5bTg mice and their wild type littermates.

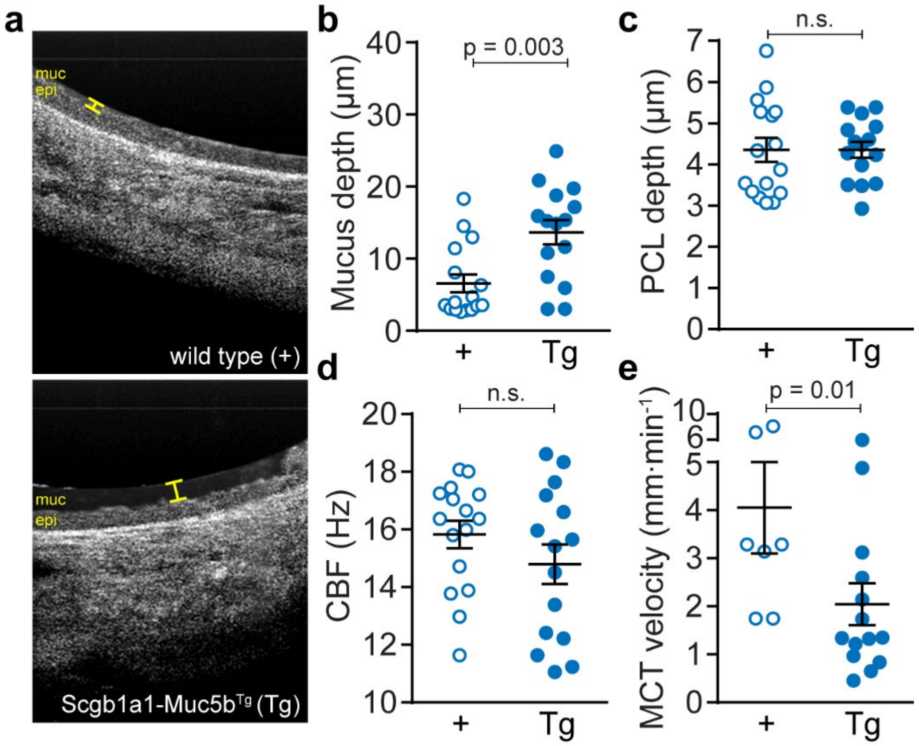

Supplementary Figure 2. Muc5b overexpression in Scgb1a1-Muc5b^{Tg} mice is associated with impaired mucociliary transport. **(a)** Representative excised Scgb1a1-Muc5b^{Tg} mice tracheas were assessed by μOCT in comparison to tracheas of wild type (+) littermate controls. **(b-e).** Quantitative metrics from image analysis reveal increased mucus layer depth **(b)** without significant alteration of periciliary layer (PCL) depth **(c)** in Tg mice compared to + controls. Functional analysis demonstrated no significant effect on ciliary beat frequency **(d)** and diminished mucociliary transport rates **(e)** in Muc5b-overexpressing mice. Data are means ± sem analyzed by Mann-Whitney test.

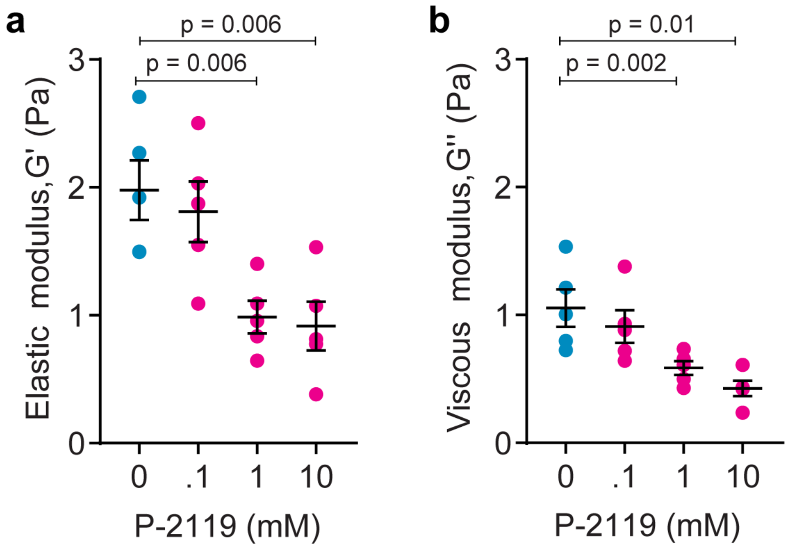

Supplementary Figure 3. Mucolytic treatment reduces elastic and viscous properties of mucus. The solid and liquid biophysical properties of viscoelastic mucus were assessed by cone-plate rheometry to determine elastic storage (G') and viscous loss (G'') moduli. **(a,b)** In 5% mucus, the storage modulus was 3-fold greater than the viscous modulus at baseline (0 mM P-2119). With 10 mM P-2119 both elastic modulus (**a**) and viscous modulus (**b**) decreased significantly. '*', $p < 0.05$between 0 and 10 mM treated samples by t-test.

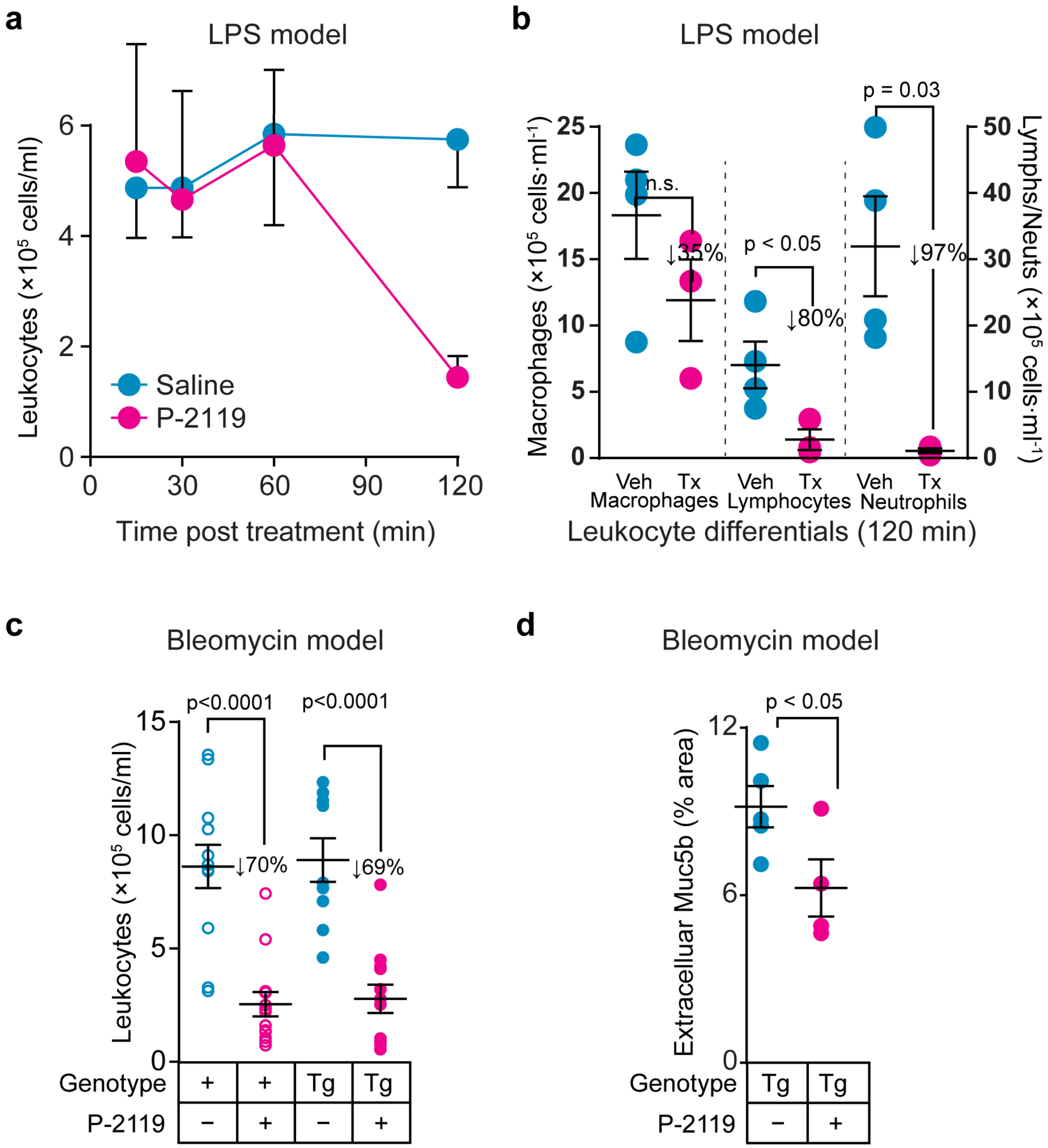

Supplementary Figure 4. Acute endogenous clearance of inflammatory cell populations in the lungs of P-2119 treated mice. (a,b) Wild type C57BL/6J mice were challenged IT with 20 µg of LPS (*E. coli* 055:B5) was administered in a 50 µl volume of saline. Mice were then treated with P-2119 or vehicle 48 h post LPS challenge. **(a)** Timecourse of leukocyte elimination in lung lavage fluid in LPS challenged mice following treatment with P-2119 aerosol (red) or saline (blue). **(b)** Leukocyte differentials in LPS challenged mice 120 min post treatment with P-2119. Lymphocyte and neutrophil numbers decreased significantly. **(c,d)** In bleomycin challenged SFTPC-Muc5b^{Tg} mice, total cells in lung lavage fluid **(c)** and extracellular Muc5b in distal airspaces **(d)** were significantly lower in P-2119 treated mice compared to vehicle treated animals. Data are means ± sem (n =3-4 mice per timepoint and treatment group). Statistical significance was determined using a t test (with Welch's correction for neutrophil numbers).

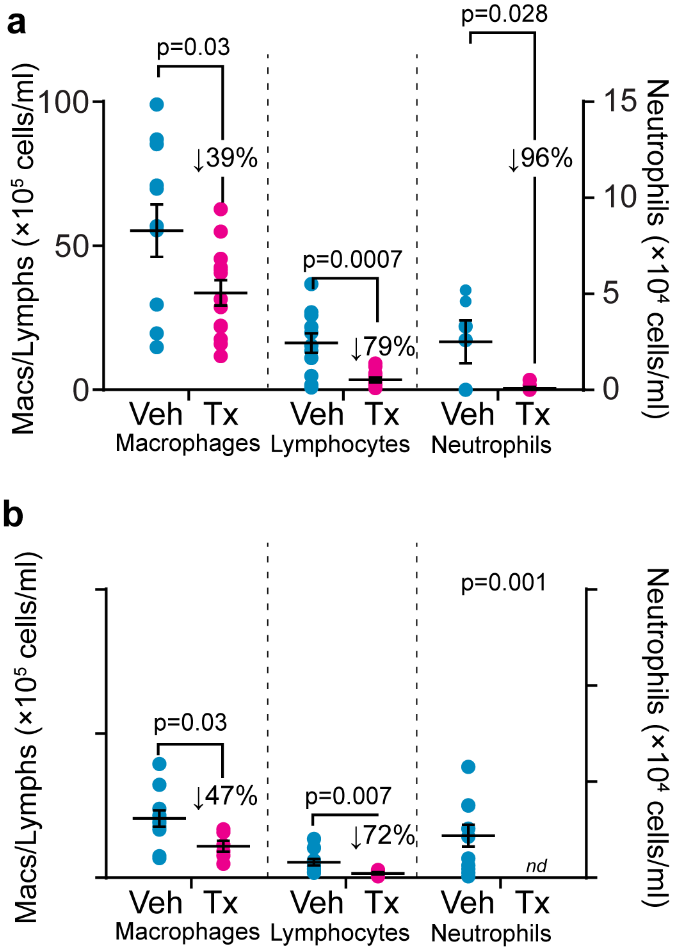

Supplementary Figure 5. P-2119 treatment reduces persistent lung injury. (**a-c**)Inflammatory cell types were enumerated in lung lavage from SFTPC-Muc5bTg mice subjected to a 3 wk (**a**) or 10 wk (**b**) bleomycin challenge and 2 wk P-2119 treatment (Tx) or saline vehicle (Veh) intervention protocol. Macrophages, lymphocytes, and neutrophils were significantly lower in in P-2119 treated mice. Data are means ± sem (n = 16 Veh and 17 Tx mice). Data were analyzed by Mann-Whitney tests.

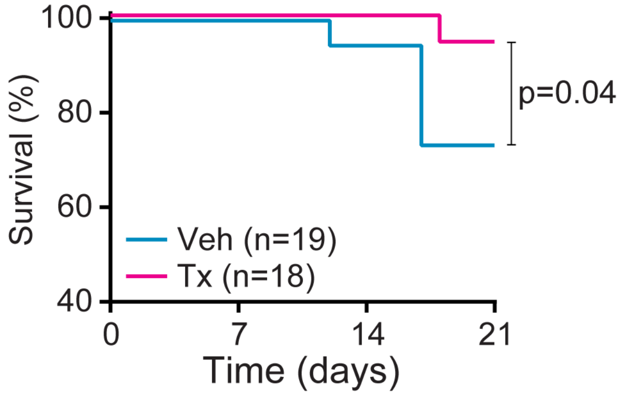

Supplementary Figure 6. P-2119 treatment results in improved survival. SFTPC-Muc5bTg mice were subjected to a 3 wk bleomycin challenge with a 2 wk P-2119 intervention protocol, with P-2119 or saline vehicle treatment starting 7 d post bleomycin challenge and ending 24 h prior to tissue harvest. Survival was improved in P-2119 treated SFTPC-Muc5b^{Tg} mice. Kaplan-Meier survival results were analyzed for significant difference by χ^2 analysis.

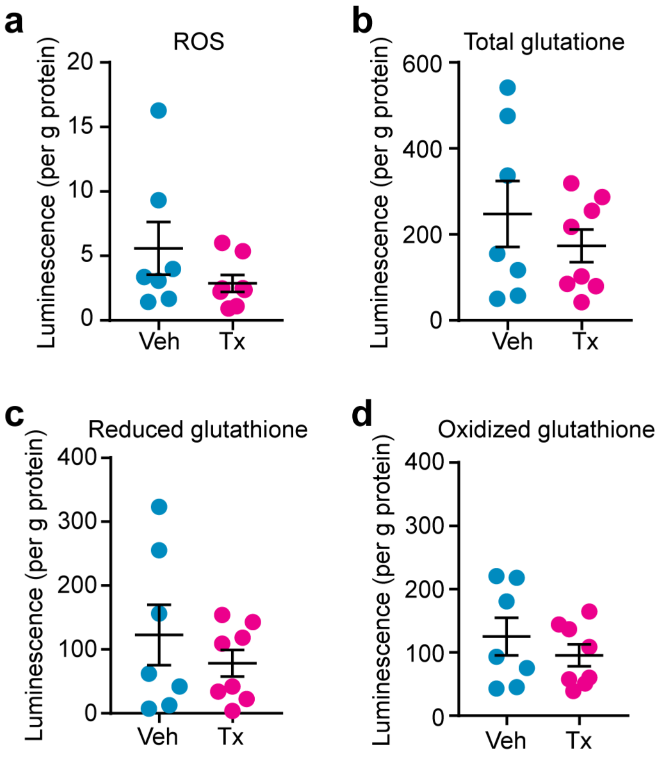

Supplementary Figure 7. P-2119 treatment of mice treated with bleomycin (3 week model) does not affect redox balance. (a) Reactive oxygen species (ROS) were assessed using ROS-GLO chemiluminescence assay in neat lung lavage obtained from mice exposed to saline vehicle (Veh) or P-2119 treatment (Tx). (**b-d**) A GSH-GLO chemiluminescence assay was used to assess total (**b**), reduced (**c**), and oxidized (**d**) glutathione. Data are means ± sem (n =7-8 mice per treatment group). Significance was assessed using a Mann-Whitney test.

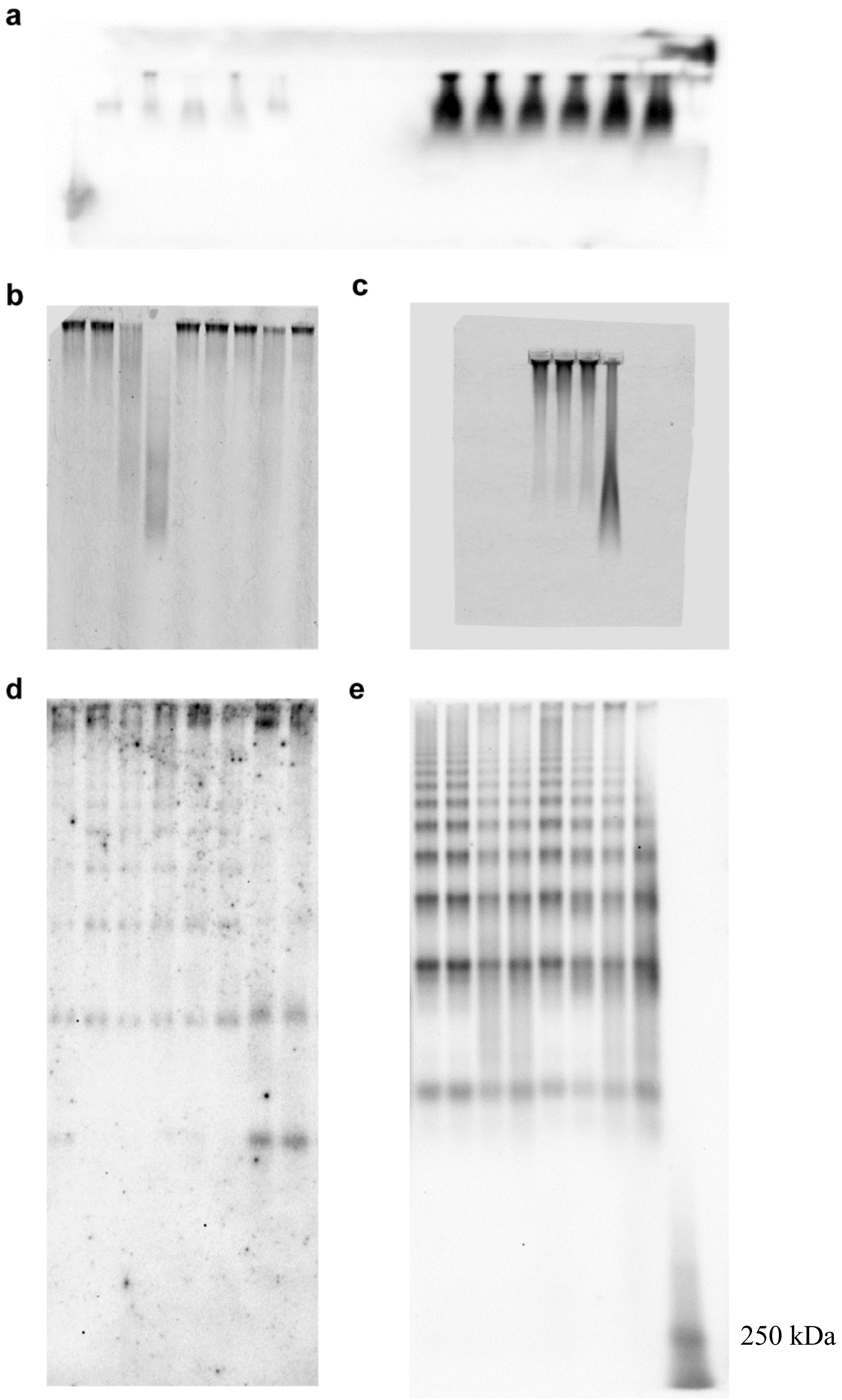

Supplementary Figure 8. Original immunoblots. Images from **Supplementary Figure 1b (a)**, **Figure 3c (b)**, **Figure 3d (c)**, **Figure 3h (d)**, and **Figure 3i (e)**. Secreted MUC5B/Muc5b monomers are >1 MDa. Rightmost lane in panel **e** is molecular weight ladder. In **a-d**, Molecular weight ladders were run to the end of gels and not transferred during vacuum blotting.

Supplementary Table 1. Cytokine and chemokine levels bleomycin challenged mice.

	Day 14 (1 week intervention)				Day 21 (2 weeks intervention)			
**Cytokine**	**Untreated (n = 8)**	**Treated (n = 9)**	**Treated Fold Δ**	**p**	**Untreated (n = 16)**	**Treated (n = 16)**	**Treated Fold Δ**	**p**
**IFN-γ**	0.0 ± 0.0	0.0 ± 0.0	1.2	n.s.	0.2 ± 0.1	0.2 ± 0.0	-1.3	n.s.
**IL-1b**	0.4 ± 0.1	0.2 ± 0.0	-1.5	n.s.	2.1 ± 0.9	1.2 ± 0.5	-1.8	n.s.
**IL-2**	0.4 ± 0.1	0.5 ± 0.1	1.2	n.s.	2.5 ± 1.1	1.4 ± 0.2	-1.8	n.s.
**IL-4**	0.2 ± 0.0	0.2 ± 0.0	1.4	n.s.	0.9 ± 0.3	0.3 ± 0.1	-2.9	n.s.
**IL-5**	1.6 ± 0.4	1.6 ± 0.2	1.0	n.s.	8.5 ± 2.5	10.2 ± 3.5	1.2	n.s.
**IL-6 ($x10^3$)**	0.1 ± 4.7	.05 ± .02	-1.9	n.s.	4.6 ± 2.3	2.6 ± 1.3	-1.7	n.s.
**IL-9**	2.1 ± 0.2	2.4 ± 0.2	1.1	n.s.	4.0 ± 1.5	2.4 ± 0.4	-1.7	n.s.
**IL-10**	0.7 ± 0.1	0.6 ± 0.1	-1.1	n.s.	9.0 ± 4.2	3.0 ± 0.6	-3.0	n.s.
**IL-12p70**	16.4 ± 2.7	23.3 ± 1.8	1.4	n.s.	135.8 ± 59.5	71.4 ± 23.6	-1.9	n.s.
**IL-15**	5.0 ± 0.3	6.6 ± 0.6	1.3	0.03	11.3 ± 1.5	10.1 ± 1.3	-1.1	n.s.
**IL-17A**	0.6 ± 0.1	0.8 ± 0.1	1.2	n.s.	1.3 ± 0.2	1.2 ± 0.1	-1.1	n.s.
**IL-30**	2.0 ± 0.3	2.0 ± 0.3	1.0	n.s.	4.8 ± 1.5	3.0 ± 0.4	-1.6	n.s.
**IL-33**	2.8 ± 0.3	2.4 ± 0.3	-1.2	n.s.	3.8 ± 0.3	4.6 ± 0.5	1.2	n.s.
**IP-10**	12.0 ± 3.4	7.9 ± 2.1	-1.5	n.s.	24.5 ± 6.0	42.6 ± 15.6	1.7	n.s.
**KC**	15.2 ± 2.6	11.0 ± 2.1	-1.4	n.s.	24.6 ± 6.4	20.4 ± 3.3	-1.2	n.s.
**MCP-1**	5.9 ± 1.5	4.7 ± 0.6	-1.3	n.s.	65.5 ± 25.8	74.5 ± 27.8	1.1	n.s.
**MIP-1α**	2.6 ± 0.8	2.3 ± 0.3	-1.2	n.s.	2.1 ± 0.2	2.3 ± 0.3	1.1	n.s.
**MIP-2**	14.6 ± 6.0	9.9 ± 5.1	-1.5	n.s.	7.6 ± 0.9	6.3 ± 0.5	-1.2	n.s.
**TNF**	20.5 ± 11.1	11.8 ± 5.9	-1.7	n.s.	7.8 ± 0.8	7.6 ± 1.1	1.0	n.s.

P-2119 treatment of SFTPC-Muc5bTg mice treated with bleomycin (2.0 U/kg on day 0, and studied on day 14 for 1 week intervention model and day 21 for 2 week intervention models) does not affect chemokine and cytokine levels. Chemokines and cytokines in lung lavage fluid were assessed using a Mesoscale 19-plex electrochemiluminescence assay from mice exposed to saline (untreated) or P-2119 treatment (treated). Data are means ± sem in pg/ml of each analyte. Statistical significance was assessed using two-tailed Mann-Whitney tests with (significance cut-off, $p < 0.05$).
