# Supplementary material for: Muc5b overexpression causes mucociliary dysfunction and enhances lung fibrosis in mice
Source: Nat Commun. 2018 Dec 18;9:5363. doi: 10.1038/s41467-018-07768-9 (PMC6299094; doi:10.1038/s41467-018-07768-9)
Supplement: Supplementary file 2 — Description of Additional Supplementary Files [file 41467_2018_7768_MOESM2_ESM.docx]

**Description of Additional Supplementary Files**

**File Name**: Supplementary video 1

**Description**: Micro-OCT example in an SFTPC-Muc5b+(wt) mouse.

**File Name:** Supplementary video 2

**Description**: Micro-OCT example in an SFTPC-Muc5bTg mouse.

**File Name**: Supplementary video 3

**Description**: Micro-OCT example in an Scgb1a1-Muc5b+(wt) mouse.

**File Name**: Supplementary video 4

**Description**: Micro-OCT example in an Scgb1a1-Muc5bTg mouse.
